# Expression and influence of KATP in umbilical artery smooth muscle cells of patients with hypertensive disorders of pregnancy

**DOI:** 10.1038/s41598-024-57885-3

**Published:** 2024-03-29

**Authors:** Benlan Yin, Xiaotong Yu, Xiaodong Fu, Xiyuan Liu, Jing Xiao, Linli Yu, Yunying Nie, Yujiao Zhang

**Affiliations:** 1 Department of Obstetrics, The Affiliated Hospital of Southwest Medical University, No.8, Kangcheng Road, Luzhou, 646000 Sichuan China; 2 Department of Clinical Medicine, Southwest Medical University, No. 319, Section 3, Zhongshan Road, Luzhou, Sichuan China

**Keywords:** KATP, Hypertensive disorders, Pregnancy, Umbilical artery, Cardiology, Diseases

## Abstract

The objective of this study is to investigate the expression and influence of adenosine triphosphate-sensitive potassium channel (KATP) in human umbilical arterial smooth muscle cells (HUASMCs) of patients with hypertensive disorders of pregnancy (HDP). Western blotting was used to detect the protein expression levels of KATP inwardly rectifying potassium channel (Kir)6.1 and sulphonylurea receptor (SUR)2B subunits in HUASMCs from patients with normal parturients (NP), gestational hypertension (GH), chronic hypertension (CH), preeclampsia (PE) and chronic hypertension with superimposed preeclampsia (CHSP), respectively. There was no significant difference in the protein expression of Kir6.1 subunit in NP group, GH group, CH group, PE group and CHSP group (*P* > 0.05). The protein expression of SUR2B subunit was gradually decreased in NP group, GH group, CH group, PE group and CHSP group, with statistically significant difference among the groups (*P* < 0.05). The altered expression level of KATP SUR2B subunit may be involved in the pathogenesis of HDP. The severity of HDP may be related to the degree of decrease of SUR2B subunit.

## Introduction

Hypertensive disorders of pregnancy (HDP) are a kind of pregnancy-specific diseases, which are classified into gestational hypertension (GH), preeclampsia (PE), eclampsia, chronic hypertension (CH), and chronic hypertension with superimposed preeclampsia (CHSP)^1^. The pathological basis of the disease is systemic small vascular spasm, accompanied by endothelial injury and ischemia, which leads to decreased blood perfusion in various organs of pregnant women, and then leads to a series of complications, which seriously threaten the health of mother and fetus. At present, the mechanism of HDP is still unclear, which may be related to the social conditions of pregnant women, pregnancy period and previous physical conditions and etc^2^.

Potassium ion channels on the membrane of arterial vascular smooth muscle cells play a major role in regulating the membrane resting potential of smooth muscle cells, and are also involved in regulating arterial tension, vasoconstriction and vascular proliferation^3^. Adenosine Triphosphate -sensitive potassium channel (KATP) consists of two distinct subunits: inwardly rectifying potassium channel (Kir) and sulphonylurea receptor (SUR) subunit. Kir is a member of the inward rectifier family, and its function is to constitute the potassium ion permeable channel in KATP. It has two isoforms, Kir6.1 and Kir6.2. SUR is a regulatory subunit and is the main target of KATP openers or inhibitors. It is divided into two subtypes, SUR1 and SUR2 (SUR2A, SUR2B and SUR2C)^4–6^.

The main roles of KATP are to couple cell metabolism and electrical activities, maintain vascular tension, regulate the function of related tissue cells, and protect organs. The composition of its channel subunits in different tissue cells is not exactly the same, and the vascular smooth muscle cells are mainly Kir6.1 and SUR2B^7^. Previous studies have shown a role for differential voltage-gated potassium channel (Kv)7 expression in the development of preeclampsia^8^. As one of the types of potassium channels, whether KATP affects the occurrence and development of HDP needs further study.

The umbilical cord is the life bridge of communication between the fetus and the placenta. A comprehensive understanding of the mechanism and structural changes of the umbilical artery in the constricted state is the basis for studying the hemorheology of the fetal-placental circulation. Therefore, the purpose of this experiment was to detect the expression and possible differences of KATP Kir6.1 and SUR2B subunit-related receptor proteins in umbilical artery smooth muscle of normal puerperae and HDP patients by Western blotting, and to explore the role of KATP in the occurrence of HDP. It is expected to have a further understanding of the pathogenesis of HDP and provide a certain basic theoretical basis for the treatment of the disease.

## Results

### Basic information of the research object

Basic information included maternal ages, body mass index(BMI), gestational ages, levels of alanine aminotransferase (ALT), aspartate transaminase (AST), platelet (PLT), creatinine, blood pressures and neonatal weight. All data was normally distributed and passed the homogeneity test. Compared with normal puerperae and HDP patients in each group, there was no significant difference in age (*P* > 0.05), BMI (*P* > 0.05), gestational days (*P* > 0.05), gestational weeks (*P* > 0.05), ALT (*P* > 0.05), AST (*P* > 0.05), PLT (*P* > 0.05), creatinine (*P* > 0.05) and neonatal weight (*P* > 0.05), and there was significant difference in blood pressure (*P* < 0.001) (Table 1).

**Table 1:** Basic information.

	NP (n = 10)	GH (n = 10)	CH (n = 10)	PE (n = 10)	CHSP (n = 10)	F	*p* values
Age (y)	31.80	30.60	29.20	31.30	30.00	0.260	0.902
BMI	22.85	22.60	20.73	22.63	22.78	1.293	0.287
Gestational days (d)	267.40	269.00	265.70	266.00	265.00	0.355	0.839
Gestational weeks (w)	38.20	38.43	37.95	38.00	37.85	0.355	0.839
ALT (U/L)	26.54	25.25	21.75	28.59	26.83	0.596	0.667
AST (U/L)	23.01	23.48	23.03	25.64	26.88	0.434	0.784
PLT (× 10^9^/L)	231.40	212.20	224.50	222.50	214.00	0.108	0.979
Creatinine (μmol/L)	54.86	54.90	59.45	55.84	56.11	0.387	0.817
Neonatal weight (kg)	3.18	2.82	3.06	2.75	2.74	1.989	0.112
SBP (mmHg)	116.00	149.00	149.10	167.90	168.10	60.703	0.000*
DBP (mmHg)	76.80	98.70	99.30	122.00	122.80	76.521	0.000*

From the above table, it can be seen that there was no significant difference in blood pressure between GH and CH group, PE and CHSP group. The difference of blood pressure among other groups was statistically significant. (*: *P* < 0.001).

## Comparative analysis of normal group and HDP groups

**Figure 1:**
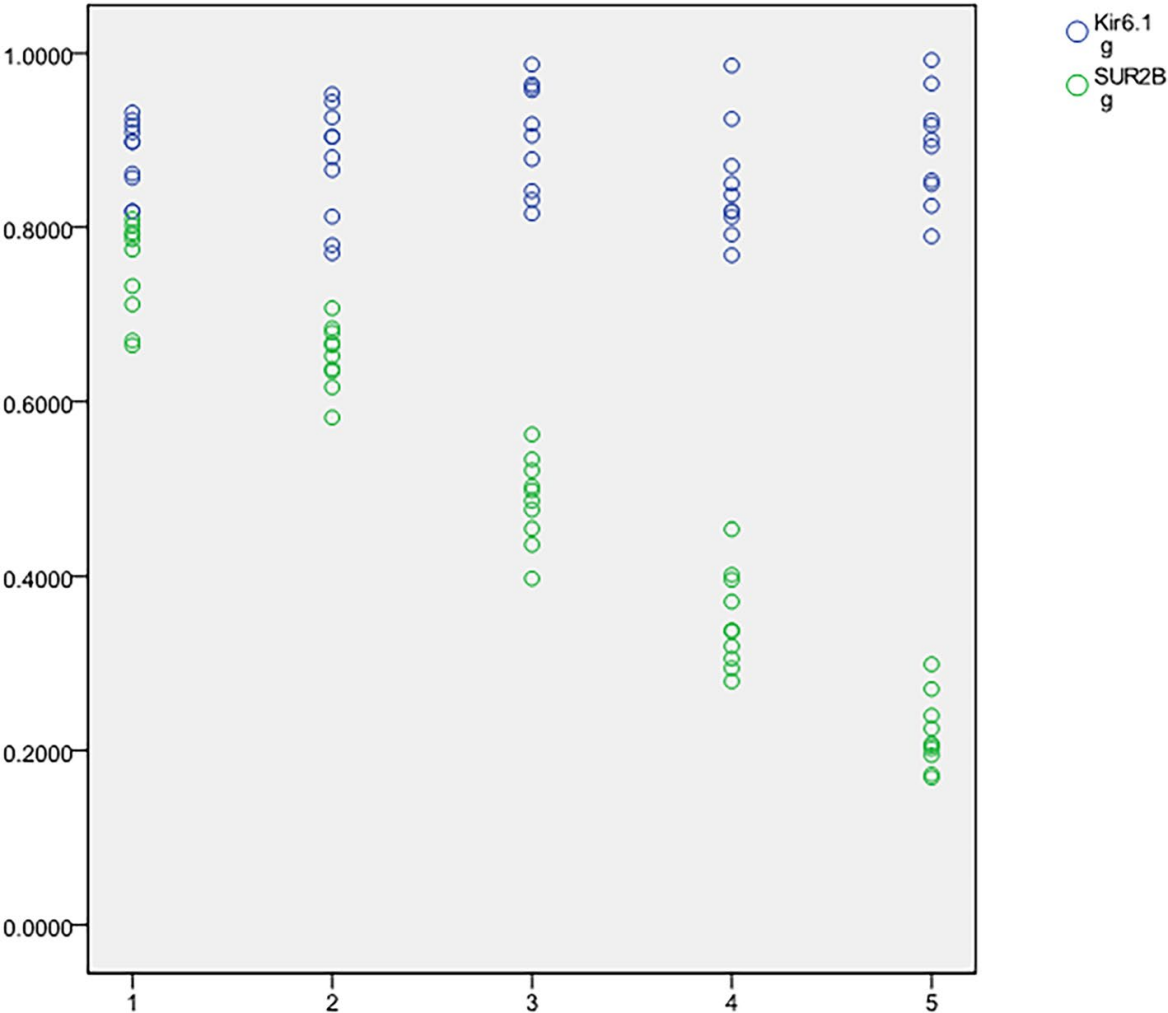
Expression of Kir6.1 and SUR2B subunit proteins in NP group and HDP groups.

**Figure 2:**
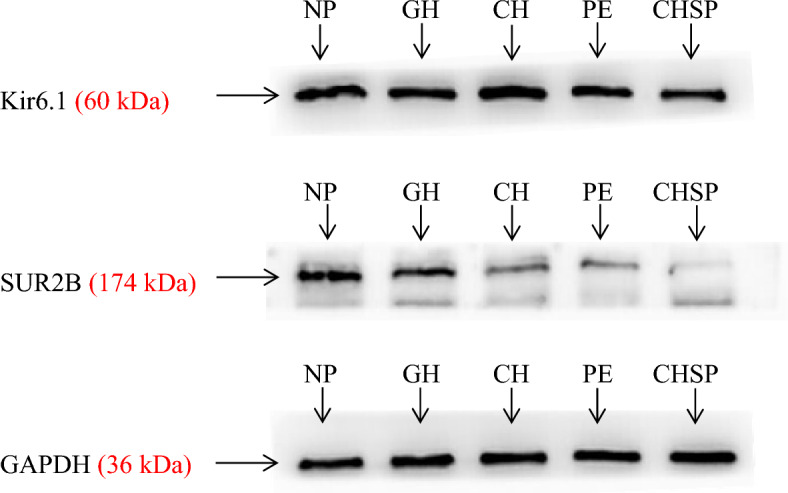
Expression imaging of Kir6.1 and SUR2B subunit proteins in NP group and HDP groups.

The gray value ratios of Kir6.1 and SUR2B subunits in Western blotting results were normally distributed in each group, and passed the homogeneity test. There was no significant difference in Kir6.1/GAPHD value among the groups (*P* = 0.282 > 0.05); however, there was a statistically significant difference in SUR2B/GAPHD value among the groups (*P* < 0.001). Specifically, the expression of SUR2B protein decreased in the NP group, GH group, CH group, PE group and CHSP group in turn. See Table 2 and Figs. 1 and 2.

**Table 2:** Comparison of Kir6.1 and SUR2B subunit protein expression differences among groups.

ANOVA						
		Sum of squares	df	Mean square	F	Sig
Kir6.1	Between groups	0.019	4	0.005	1.306	0.282
	Within groups	0.162	45	0.004		
	Total	0.181	49			
SUR2B	Between groups	1.893	4	0.473	206.863	0.000*
	Within groups	0.103	45	0.002		
	Total	1.996	49			

There was no significant difference in the expression of Kir6.1 among the groups (*P* = 0.282 > 0.05), but there was a significant difference in the expression of SUR2B among the groups (*: *P* < 0.001).

## Discussion

This paper explores the expression and influence of KATP in HDP patients. The results of Western blotting in this study showed that compared with normal puerperae, the expression of KATP SUR2B subunit in umbilical artery smooth muscle cells of HDP patients was significantly reduced, and the degree of reduction in different types was not consistent. The more severe the type of symptoms, the more obvious the reduction. However, there was no significant difference in the expression of Kir6.1 subunits among several groups.

Since the Kir6.1 subunit acts as a pore for potassium ion permeability in KATP, it is a structural subunit; SUR is the main target of KATP openers or inhibitors, and is a regulatory subunit. Therefore, it is speculated that the decreased expression of SUR2B subunit of KATP is related to the occurrence and development of HDP, and its abnormal expression may be the reason for the diastolic and systolic dysfunction of umbilical artery smooth muscle cells in patients with HDP. And the lower the expression of SUR2B subunit, the more severe the clinical symptoms of patients may be. So what about the relationship between KATP and other cardiovascular and cerebrovascular diseases and the expression of other potassium channels in HDP?

About the KATP and cardiovascular and cerebrovascular diseases, the study by Flori et al.^9^ showed that erucin cardioprotective effects against ischemia/reperfusion (I/R) damage through the involvement of mito-KATP and the consequent depolarizing effect, which in turn reduced calcium entry and preserved mitochondrial integrity. In view of this characteristic, there have been a lot of studies on KATP openers in recent years, and it has been proved that KATP openers can play a cardioprotective role in many species, and are widely used in myocardial ischemia, myocardial hypertrophy and other fields^10^, which is consistent with our research findings.

KATP is widely distributed in the brain area. During cerebral infarction, the human body is in a pathological state of hypoxia or insufficient nutrient delivery, ATP levels are reduced, and KATP is activated, which in turn can inhibit apoptosis, reduce excitotoxicity, reduce calcium overload and regulate oxidative stress to reduce damage^11,12^. It is suggested that KATP may be an important therapeutic target for the treatment of cerebral infarction. Nicorandil, whose chemical name is N-(2-hydroxyethyl)-nicotinamide nitrate, is the first KATP opener used in clinic. Studies have shown that nicorandil can improve vascular dementia and Huntington's disease^13,14^. Studies have also shown that nicorandil treatment could decrease brain damage, improve learning and memory^15^.

Another scholar reported that KATP opener can improve endothelial colony-forming cells’ function in hypoxia via Akt/endothelial nitric oxide synthase pathways, which may constitute increase endothelial colony-forming cells’ therapeutic potential for hypoxia-associated pulmonary hypertension treatment^16^. Nikbakht et al^17^ confirmed through the regulation of mitochondrial dynamic proteins that mitoKATP has an important role in balancing mitochondrial dynamic proteins in epilepsy. Specterman et al^18^ found that KATP block/absence leads to cellular and tissue level atrial electrophysiological modification. Kir6.2 global knockout prevents hypoxia-induced atrial wavefront path length shortening and atrial arrhythmogenicity to programmed electrical stimulation.

So, it can be seen that KATP plays an important role in the occurrence, development and treatment of various cardiovascular and cerebrovascular diseases, which is consistent with our direction of results.

And about potassium channels and HDP, some studies have suggest a role for differential placental Kv7 expression in the development of preeclampsia. Functional studies are needed to determine processes affected by Kv7 in the placenta ^8^. Large-conductance calcium-activated potassium channels (BKCa) are a major type of potassium channel. Our previous study found through QPCR and Western blotting that compared with normal women, the expression level of β1 subunit in the BKCa of placental arterioles, uterine arterioles and mesenteric artery smooth muscle cells in HDP patients decreased, but the expression of α subunit did not change significantly, suggesting that the abnormal expression of β1 subunit may be an important basis for vasomotor dysfunction in HDP patients. In addition, through single-channel patch-clamp study, we found that compared with normal women, the current amplitude of BKCa in uterine arterioles and placental arterioles smooth muscle cells in patients with severe preeclampsia decreased, the opening probability was reduced, and the average opening time was shortened, indicating that the functional activity of BKCa in uterine and placental arteriole smooth muscle was impaired or weakened in patients with severe preeclampsia. BKCa may be an important regulatory node in the occurrence and development of severe preeclampsia. So, it is speculated that KATP, as another type of potassium channel, may also be involved in the occurrence of HDP. In the study of other vascular diseases, abnormal changes of KATP can cause hypertension, and KATP opener can improve related clinical symptoms. This also matches the results of this experiment.

Therefore, we speculate that KATP opener can improve the function of HDP umbilical artery vascular smooth muscle and induce vasodilation by increasing the expression of SUR2B subunit, thereby improving fetal blood supply and reducing the occurrence of intrauterine growth restriction or intrauterine stillbirth.

## Conclusions

The Kir6.1 subunit is a structural subunit and the SUR2B subunit is a functional subunit. Our results suggest that the abnormal expression of KATP SUR2B subunit in HUASMCs may be involved in the occurrence of HDP, and the lower the expression of SUR2B subunit, the more severe the clinical symptoms may be in HDP patients. But there is no research to explore relevant monitoring indicators to prevent the occurrence of the disease. Its openers, such as binadil, nicandil, etc., have not yet been used for clinical treatment of HDP.

Therefore, in follow-up studies, we will set up a negative control group and a positive control group to explore the effects of KATP specific openers and blockers on channel diastolic and contraction functions using patch clamp technology, and further confirm the role of potassium channel changes in the occurrence and development of HDP. Considering the changes in the placenta and umbilical cord are not necessarily changes in the mother, we will pay more attention to the situation of the mother organism itself. On this basis, further research can be carried out through animal experiments to develop relevant targeted drugs, and clinical trials can be conducted to determine their therapeutic effects after confirming the safety of the drugs. It is also possible to study the related predictors of the SUR2B subunit of the KATP to provide a basis for preventing the occurrence of HDP. It is expected that these studies will eventually be applied in the clinic to control the occurrence and development of HDP, thereby improving maternal and fetal prognosis.

## Materials and methods

### Sample collection and patient enrollment

Research objects: HDP patients of reproductive age admitted to the Obstetrics Department of the Affiliated Hospital of Southwest Medical University from January 2022 to June 2023 and normal puerperae admitted to the hospital during the same period. The diagnostic criteria are based on the current 9th edition of Obstetrics and Gynecology in China (edited by Xing Xie, Beihua Kong, Tao Duan)^1^. GH: Hypertension occurs after 20 weeks of pregnancy, with systolic blood pressure ≥ 140 mmHg or diastolic blood pressure ≥ 90 mmHg, and returns to normal within 12 weeks postpartum; Urinary protein (−). CH: Systolic blood pressure ≥ 140 mmHg or diastolic blood pressure ≥ 90 mmHg occurs before 20 weeks of pregnancy, with no significant worsening during pregnancy. PE: systolic blood pressure ≥ 140 mmHg or diastolic blood pressure ≥ 90 mmHg occurs after 20 weeks of pregnancy, accompanied by urinary protein ≥ 0.3 g/24 h; or, although there is no proteinuria, any of the following conditions are present: platelet count < 100 000/μL; transaminases more than twice the normal value; creatinine ≥ 1.1 mg/dL; pulmonary edema; new neurological abnormalities. CHSP: Women with chronic hypertension have no proteinuria before pregnancy, but proteinuria occurs after 20 weeks of pregnancy; or hypertensive women have proteinuria before pregnancy, but after pregnancy, there is a significant increase in proteinuria, or further elevation of blood pressure, or platelet count < 100 000/μL, or liver and kidney dysfunction, pulmonary edema, etc. Inclusion criteria: those who meet the above diagnosis will be included in the experiment. Excluding multiple pregnancy, single umbilical artery, combined medical and surgical diseases and infectious diseases, combined with other pregnancy-specific diseases and obstetric complications: gestational diabetes mellitus, intrahepatic cholestasis of pregnancy, placenta previa, placental abruption, etc.

The experimental materials were the umbilical artery specimens of the parturients who met the above inclusion criteria. A total of 50 samples were included in the study: 10 normal puerperae (NP), 10 GH patients, 10 CH patients, 10 PE patients, and 10 CHSP patients admitted during the same period. The sample size is determined by the number of HDP patients received by our hospital each year and the sample number available for statistical analysis.

### Ethics approval and consent to participate

All methods were carried out in accordance with the Declaration of Helsinki and this study has been approved by the ethics committee of the Affiliated Hospital of Southwest Medical University, and all pregnant women were informed about the postpartum specimen collection and signed the corresponding consent form.

### Specimen collection, isolation, and cryopreservation

After termination of pregnancy in all cases that met the inclusion criteria, when the placenta was delivered, a 4 cm-long umbilical cord specimen was immediately taken under sterile conditions, put into a specimen box, placed in a 4 °C portable refrigerator, and immediately transferred to the laboratory. Placed the umbilical cord in pre-chilled enzyme-free water, separated the umbilical artery on ice, cut along the longitudinal axis, scraped gently with tweezers to remove endothelial cells, placed in a cryopreservation tube, marked accordingly, frozen in liquid nitrogen and transferred to −80 ℃ ultra-low temperature refrigerator for Western blotting detection.

### Western blotting experiment

We took out the specimens from each group, ground the tissue into powder under liquid nitrogen, added RIPA protein lysate containing protease inhibitor PMSF, disrupted the tissue cells by an ultrasonic disruptor to release tissue proteins, centrifuged to take the supernatant, added protein loading buffer, then heated to denature proteins and stored at −20 °C. Prepared 8% separating gel and 5% stacking gel according to the appropriate ratio, added samples to the gel wells for electrophoresis, transfered to PVDF membrane, blocked in 5% skim milk for 2 h, rinsed with phosphate buffer saline and Tween (PBST), added the primary antibody and incubated overnight on a shaker. In order to reduce interference caused by polyclonal antibodies and save resources, the blots were cut prior to hybridisation with antibodies. The primary antibody concentration was GAPDH (Bioworld, China) 1:10,000, Kir6.1 (Alomone, Israel) 1:400, SUR2B (Abcam, UK) 1:500. The primary antibody of Kir6.1 and SUR2B were polyclonal antibodies with predicted molecular weights of 60 kDa and 174 kDa, respectively. The primary antibody of GAPDH was monoclonal antibody with predicted molecular weight of 36 kDa. The membrane was washed with PBST and incubated with secondary antibody for 2 h. Preparation concentration: GAPDH secondary antibody was 1:10,000 Goat anti-Mouse IgG(H&L)-HRP (Bioworld, China). Kir6.1 and SUR2B secondary antibodies were 1:10,000 Goat anti-Rabbit IgG Secondary Antibody (Millipore, USA). After washing the membrane with PBST, placed it in the imager and added luminescent solution for exposure, imaged and saved the band diagram.

### Statistical analysis of experimental results

Quantity-One was used to analyze the gray scale of the band diagram obtained by the Western blotting experiment. By comparing with the internal reference protein GAPDH, the relative expression levels of Kir6.1 and SUR2B of the target proteins were obtained, that is, the gray value ratio. For the comparison of sample size among multiple groups, we used one-way ANOVA. The obtained data were analyzed by SPSS 24.0, and the difference was considered to be statistically significant if the result was *P* < 0.05. Figure 2 provides a set of protein band images. All original images can be found as Supplementary Information 1 and 2 online.

### Supplementary Information

Supplementary Information 1.

Supplementary Information 2.

## Data Availability

Data is provided within the manuscript or supplementary information files.

## Abbreviations

KATP: Adenosine triphosphate-sensitive potassium channel
HUASMCs: Human umbilical arterial smooth muscle cells
HDP: Hypertensive disorders of pregnancy
Kir: Inwardly rectifying potassium channel
SUR: Sulphonylurea receptor
NP: Normal parturients
GH: Gestational hypertension
CH: Chronic hypertension
PE: Preeclampsia
CHSP: Chronic hypertension with superimposed preeclampsia
Kv: Voltage-gated potassium channel
BMI: Body mass index
ALT: Alanine aminotransferase
AST: Aspartate transaminase
PLT: Platelet
BKCa: Large-conductance calcium-activated potassium channels
PBST: Phosphate buffer saline and Tween
