# Supplementary material for: Expression and influence of KATP in umbilical artery smooth muscle cells of patients with hypertensive disorders of pregnancy
Source: Sci Rep. 2024 Mar 29;14:7517. doi: 10.1038/s41598-024-57885-3 (PMC10980746; doi:10.1038/s41598-024-57885-3)
Supplement: Supplementary file 1 — Supplementary Information 1. [file 41598_2024_57885_MOESM1_ESM.pdf]

All blots are original images and have never been cropped. Because the blots are cut prior to hybridisation with antibodies in order to reduce interference caused by polyclonal antibodies and save resources.

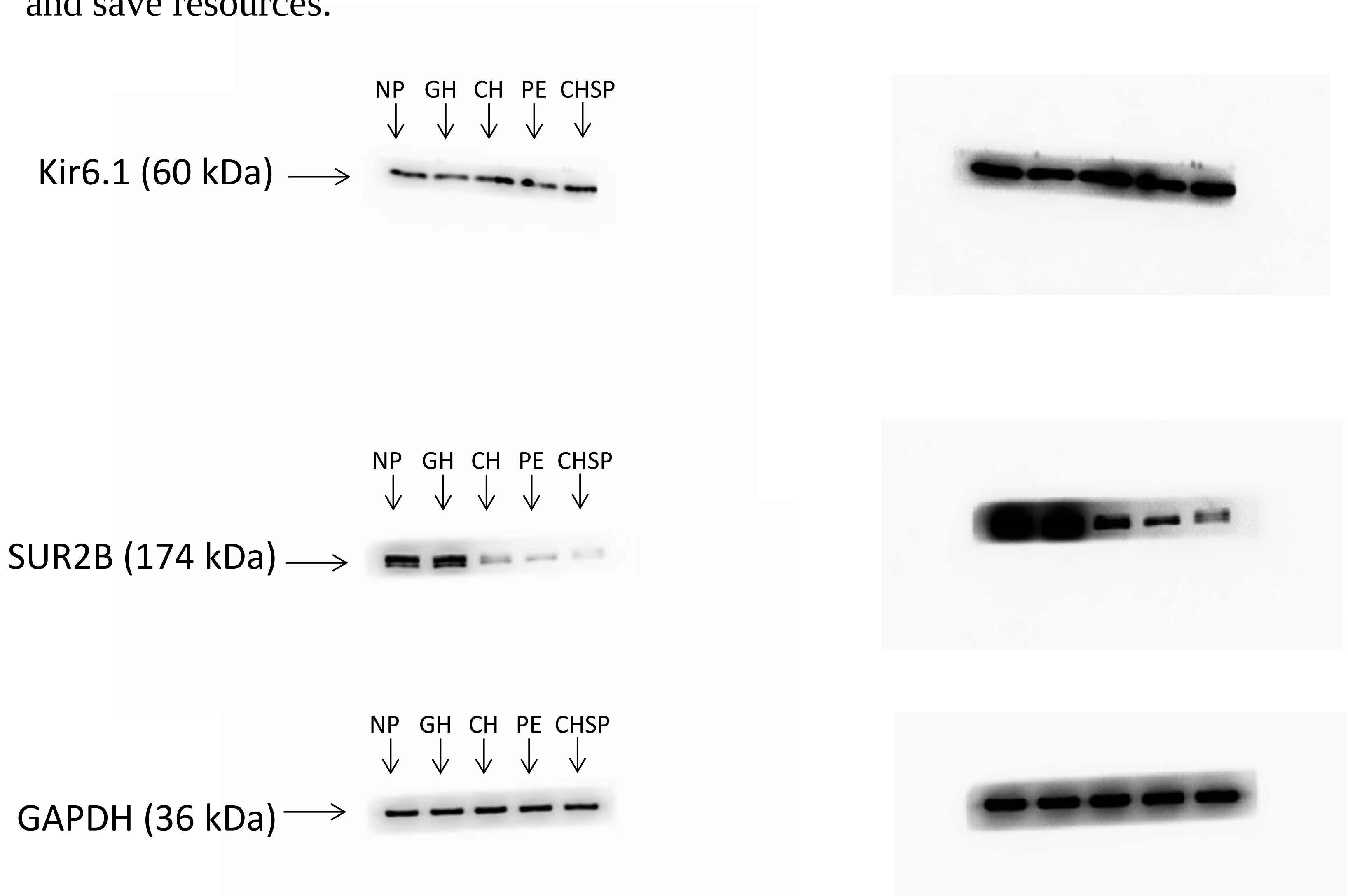

Fig. 1 Protein expression of the first group of samples.
On the right are the same blot images with membrane edges visible after adjusting through Quantity-One.

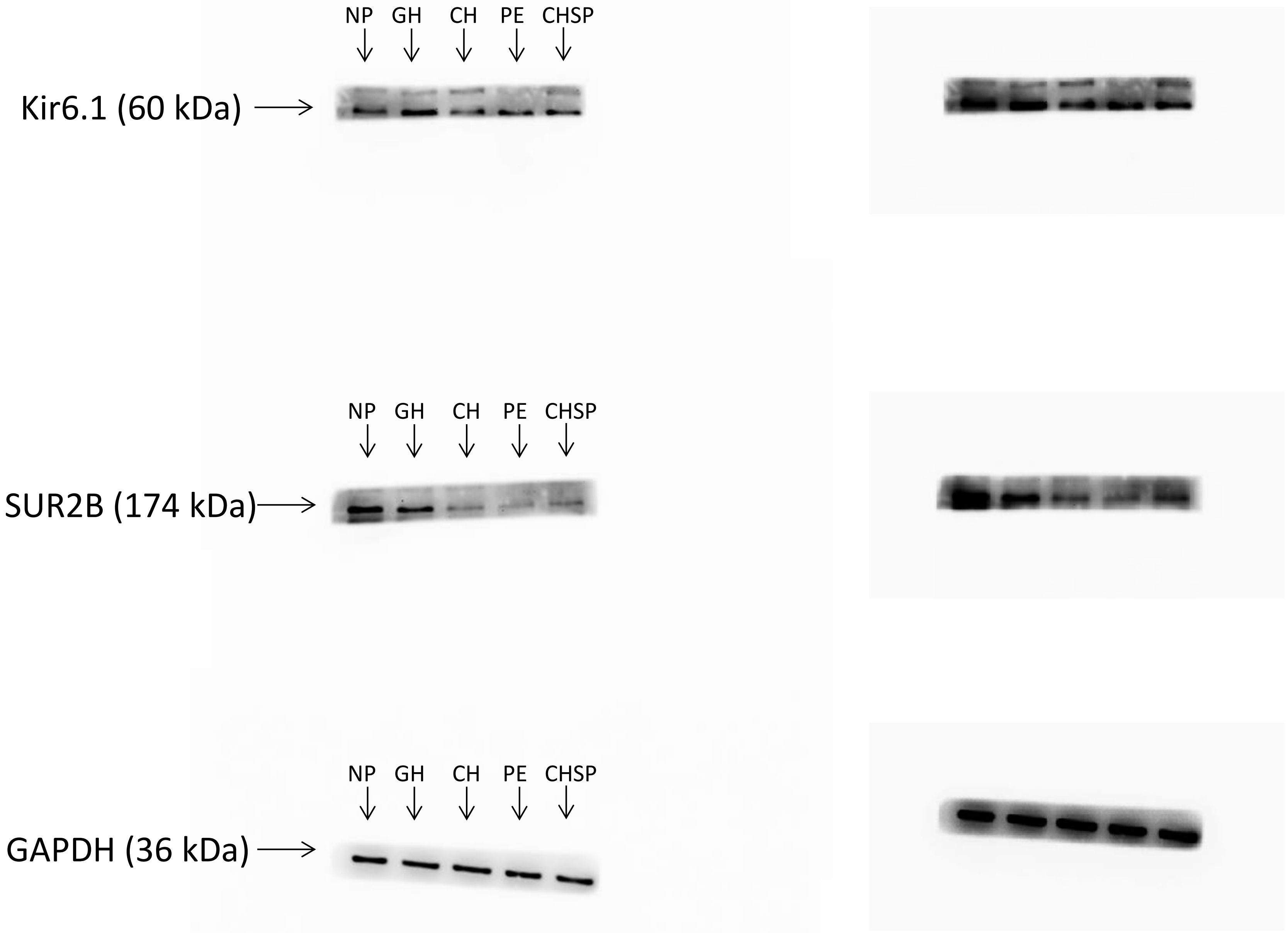

Fig. 2 Protein expression of the second group of samples. On the right are the same blot images with membrane edges visible after adjusting through Quantity-One.

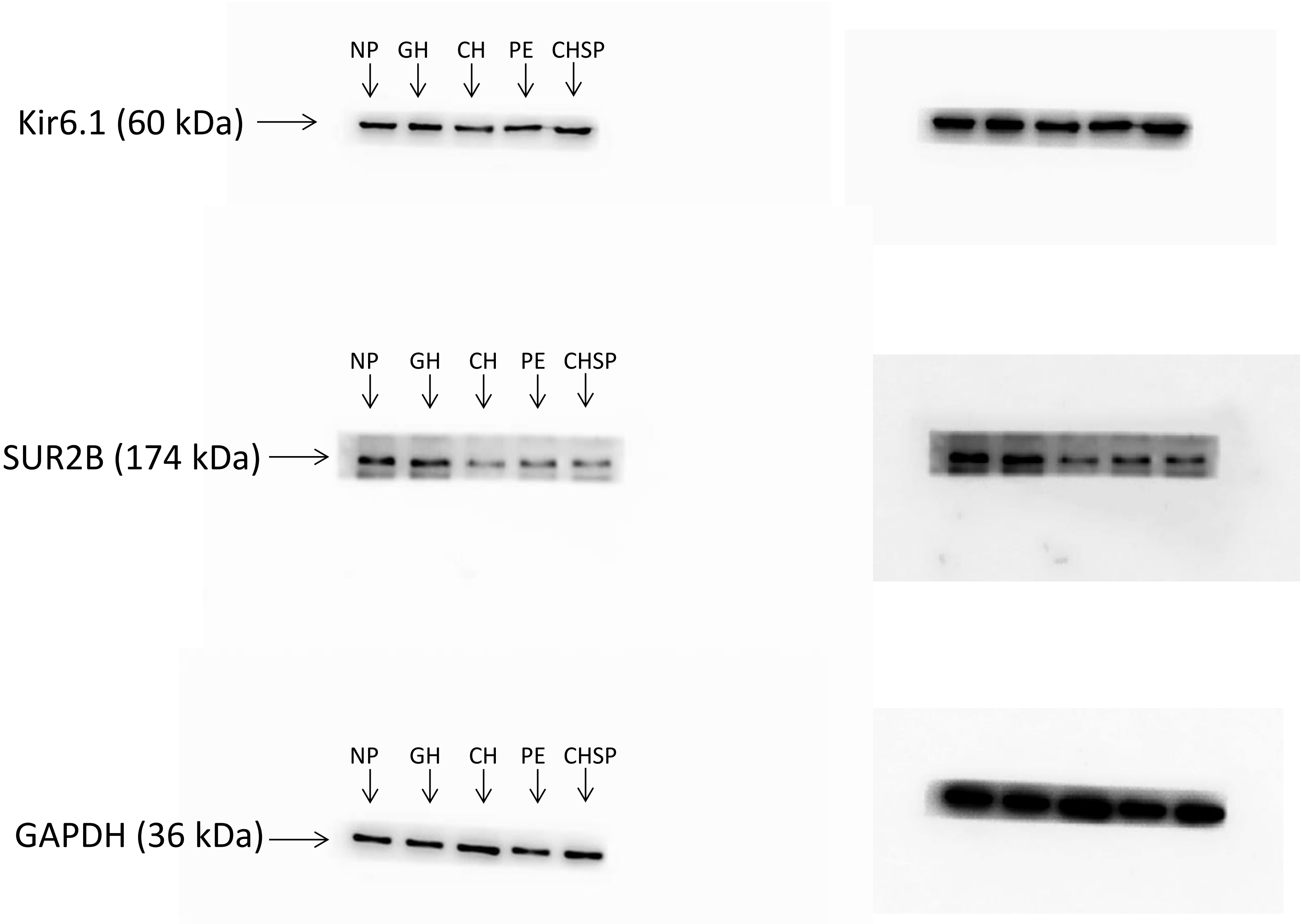

Fig. 3 Protein expression of the third group of samples. On the right are the same blot images with membrane edges visible after adjusting through Quantity-One.

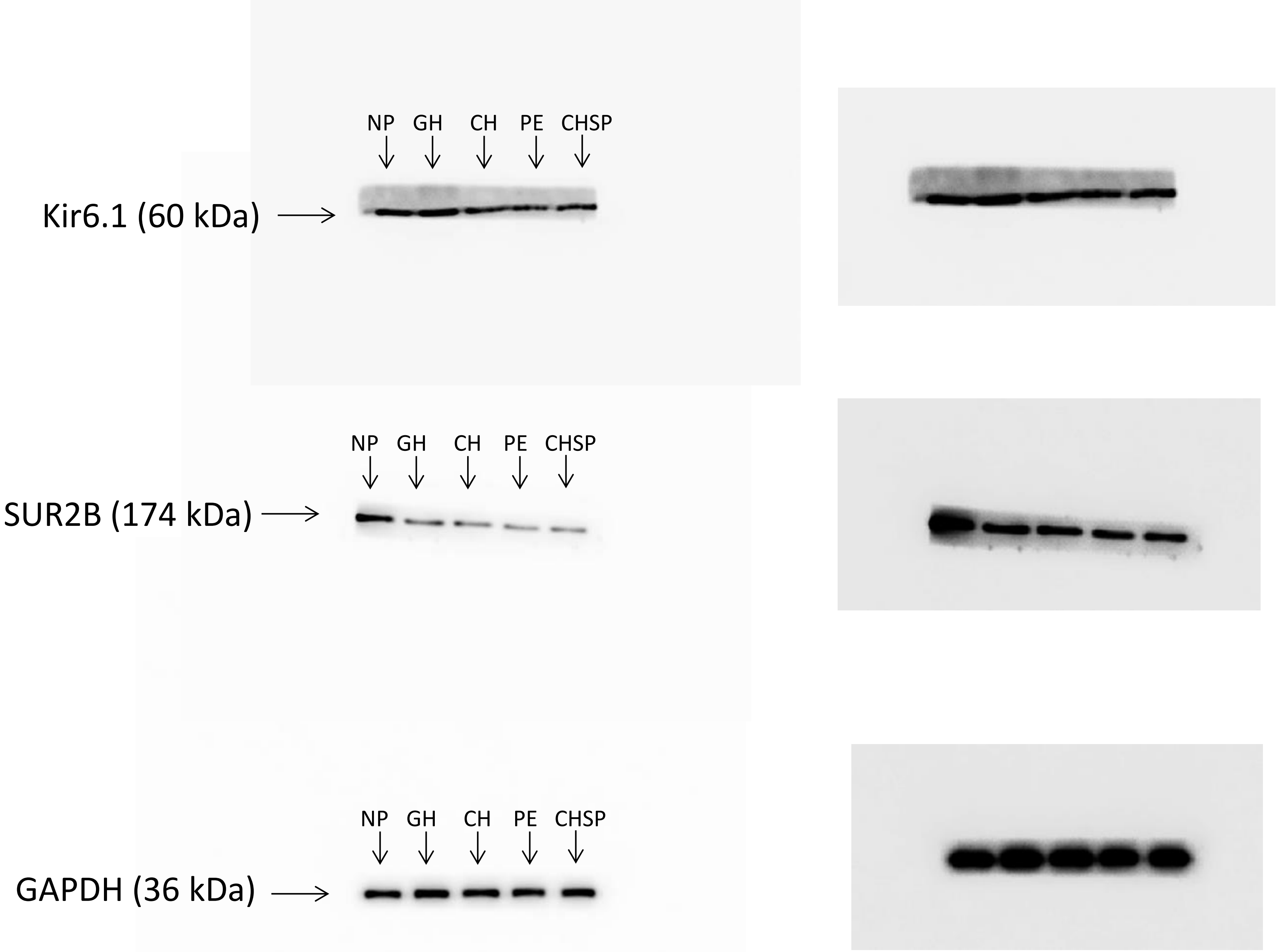

Fig. 4 Protein expression of the fourth group of samples. On the right are the same blot images with membrane edges visible after adjusting through Quantity-One.

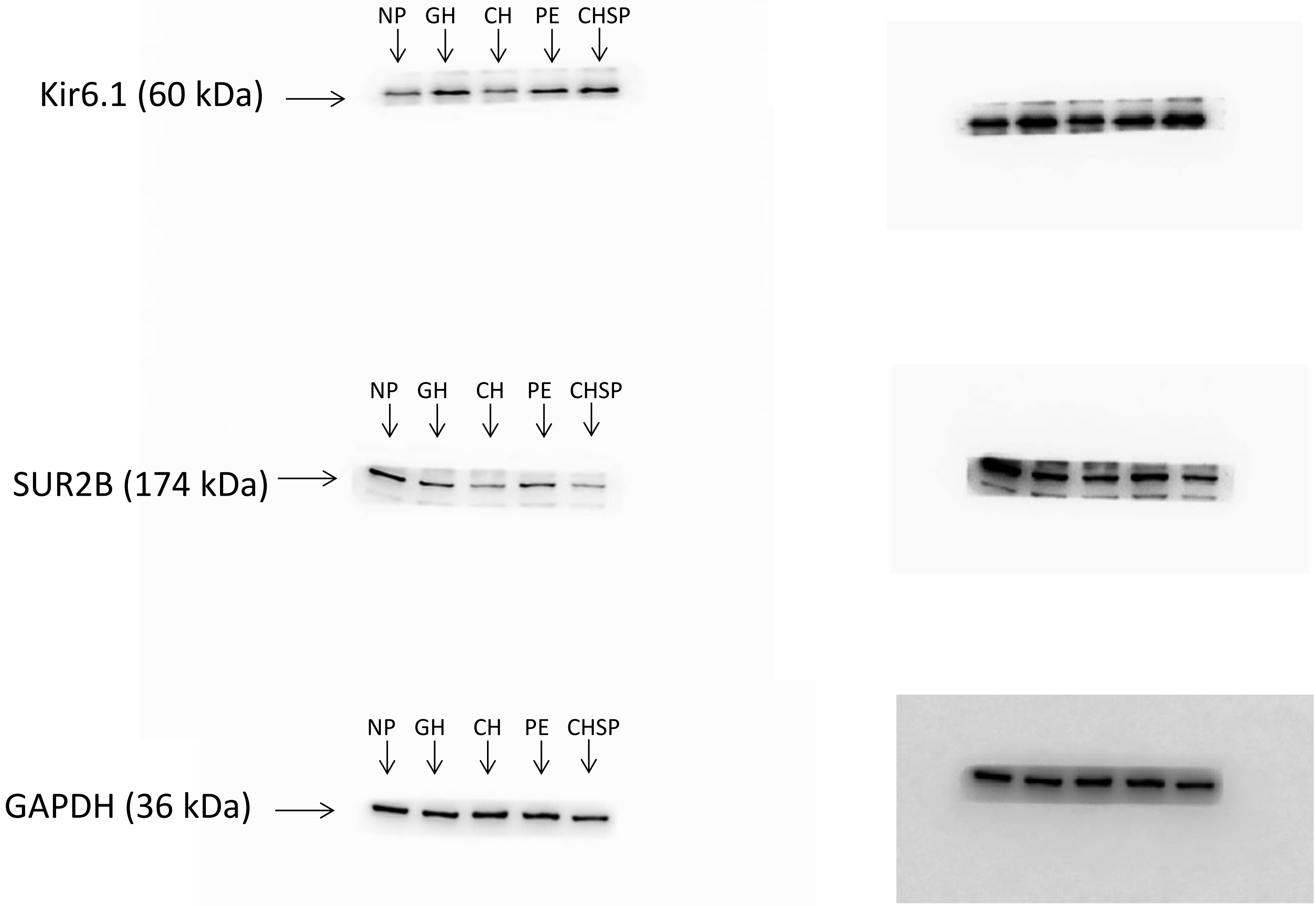

Fig. 5 Protein expression of the fifth group of samples. On the right are the same blot images with membrane edges visible after adjusting through Quantity-One.

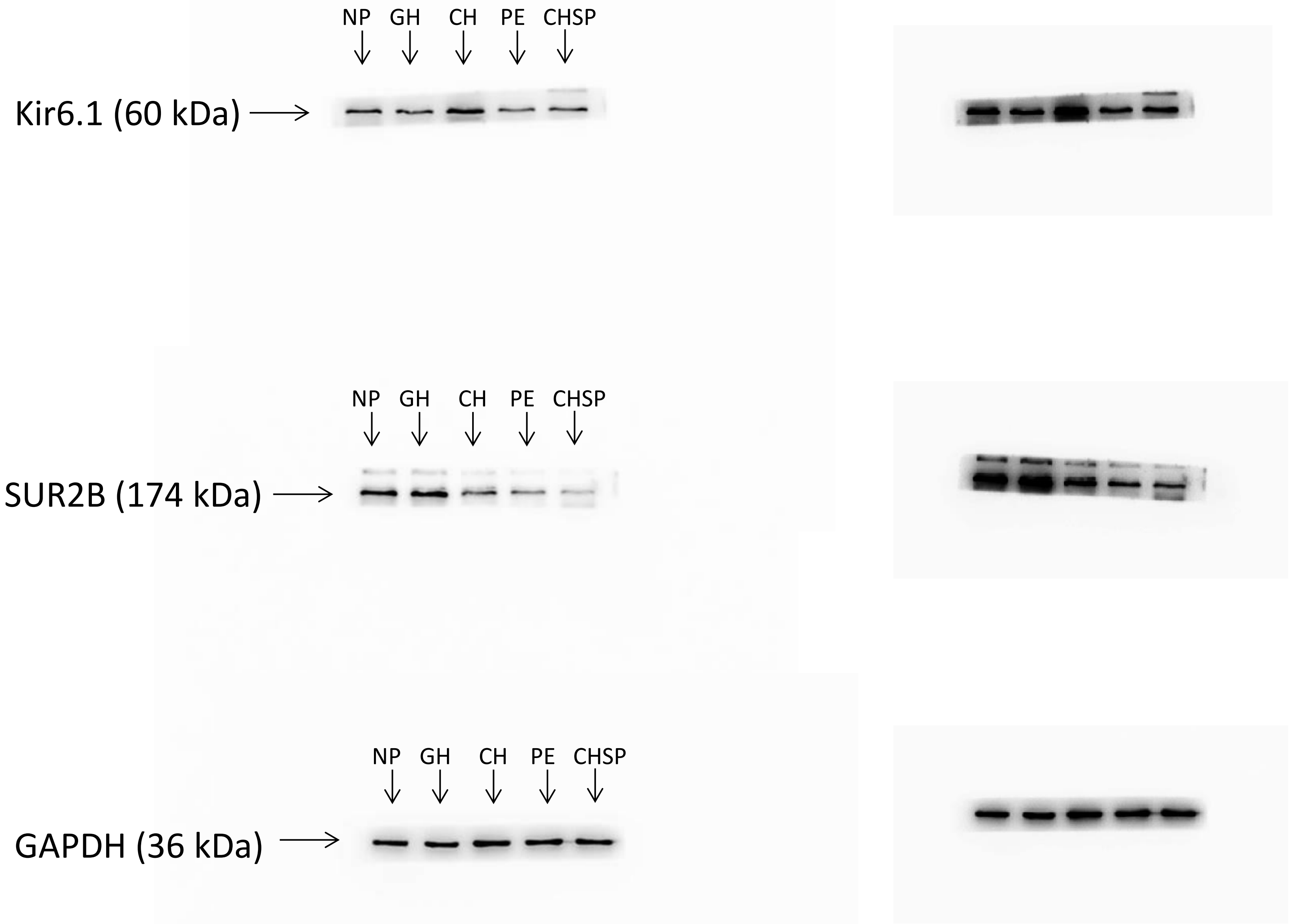

Fig. 6 Protein expression of the sixth group of samples. On the right are the same blot images with membrane edges visible after adjusting through Quantity-One.

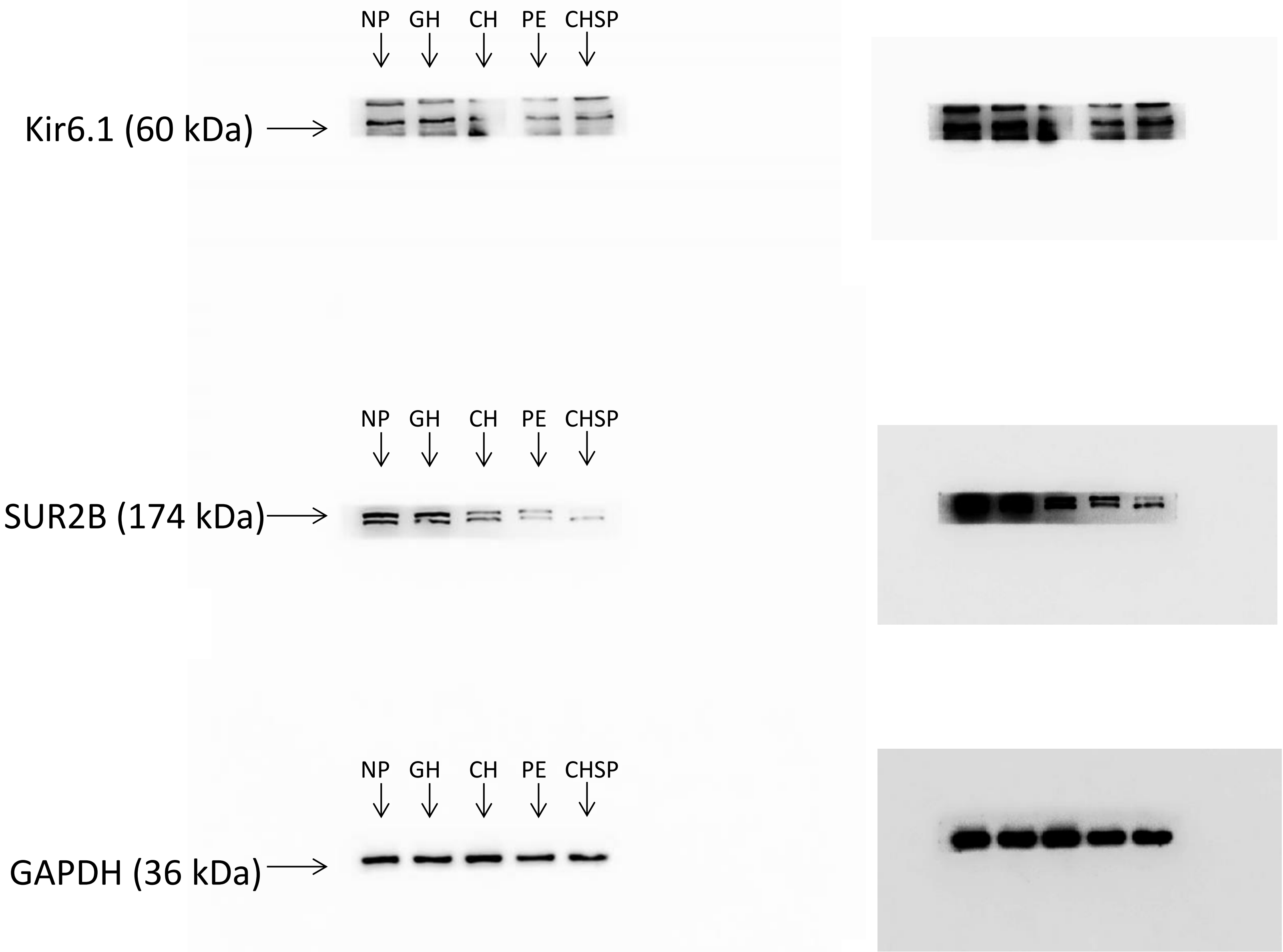

Fig. 7 Protein expression of the seventh group of samples. On the right are the same blot images with membrane edges visible after adjusting through Quantity-One.

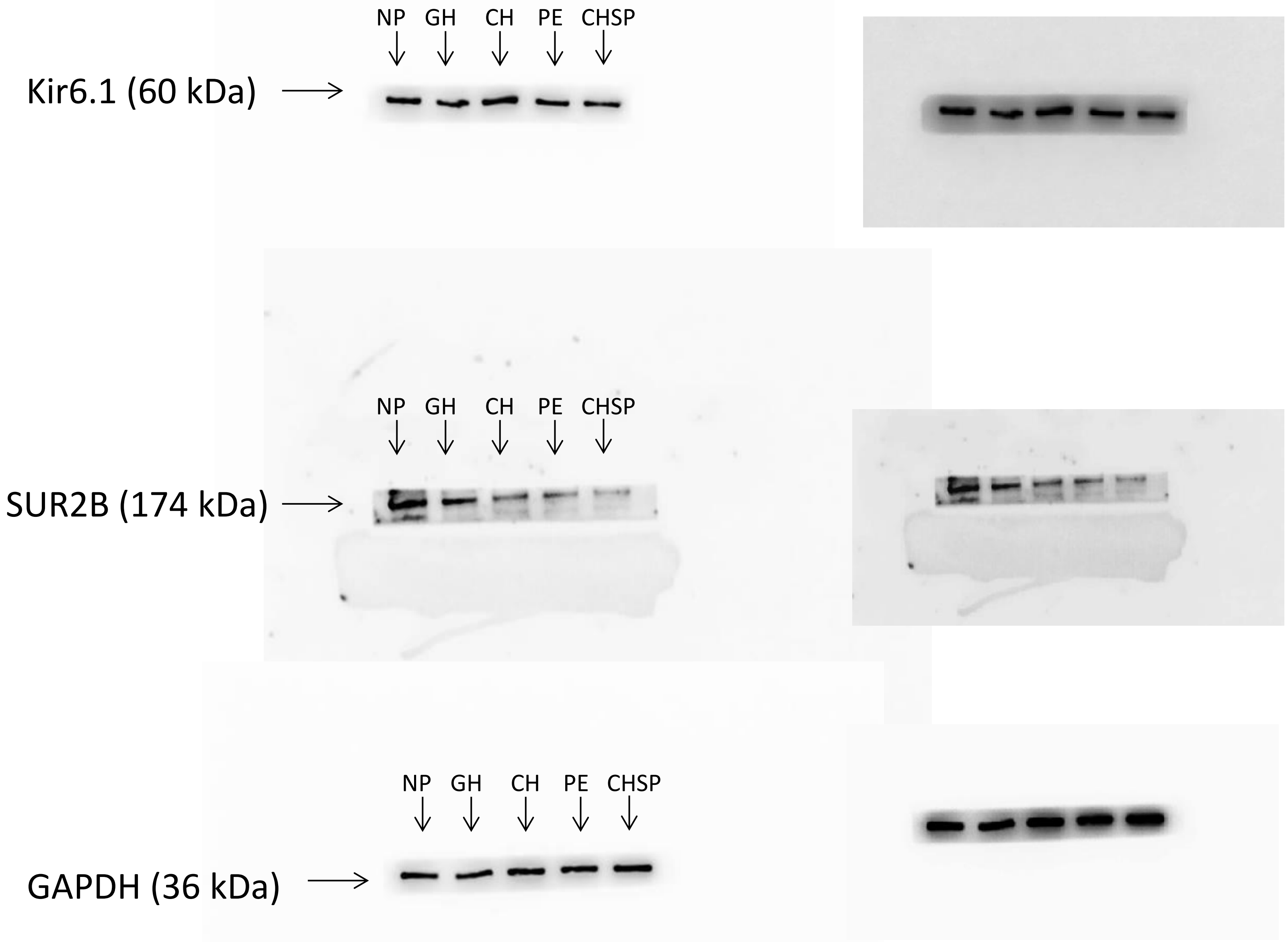

Fig. 8 Protein expression of the eighth group of samples. On the right are the same blot images with membrane edges visible after adjusting through Quantity-One.

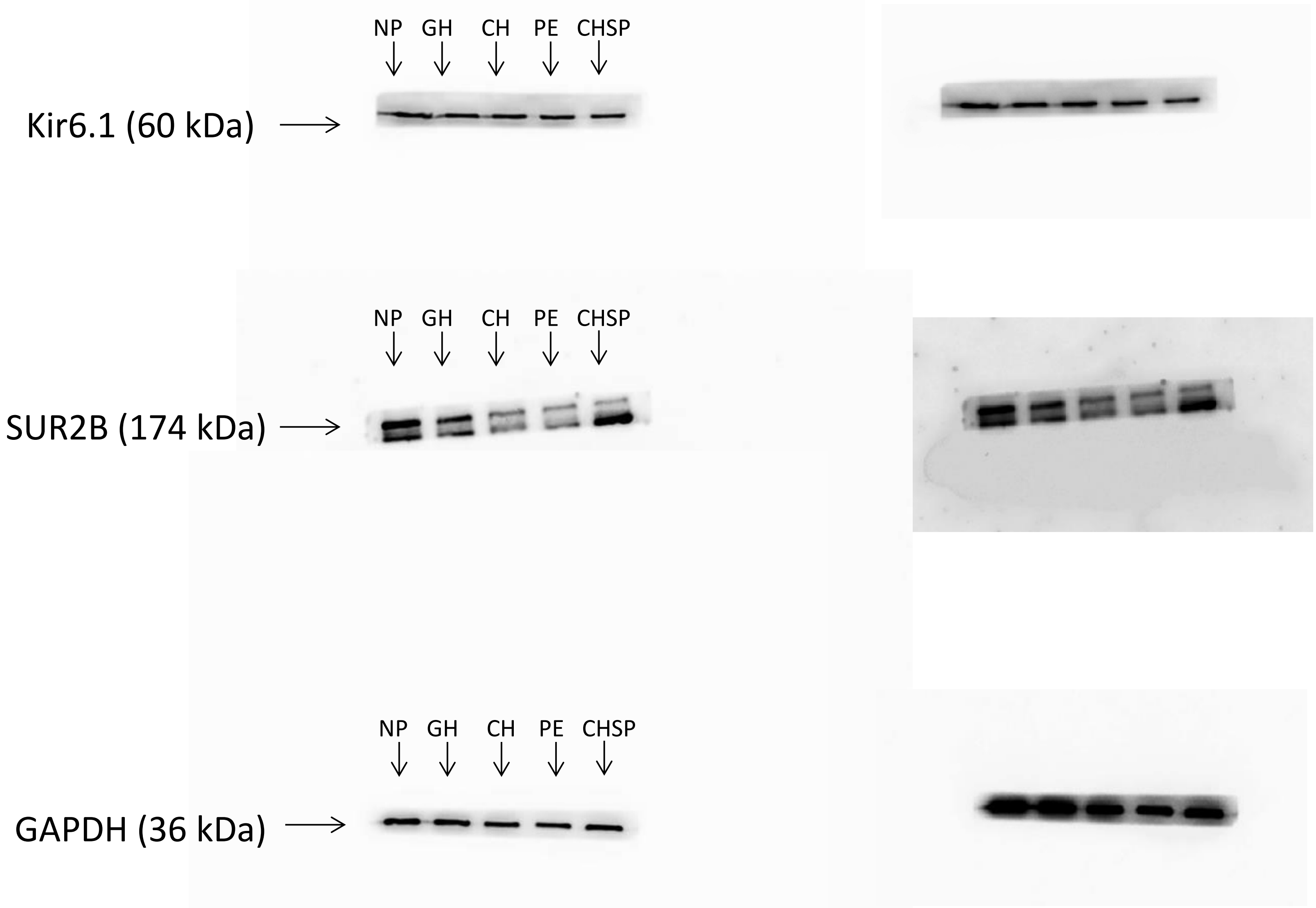

Fig. 9 Protein expression of the ninth group of samples. On the right are the same blot images with membrane edges visible after adjusting through Quantity-One.
