# Supplementary material for: Expression and influence of KATP in umbilical artery smooth muscle cells of patients with hypertensive disorders of pregnancy
Source: Sci Rep. 2024 Mar 29;14:7517. doi: 10.1038/s41598-024-57885-3 (PMC10980746; doi:10.1038/s41598-024-57885-3)
Supplement: Supplementary file 2 — Supplementary Information 2. [file 41598_2024_57885_MOESM2_ESM.docx]

All blots are original images and have never been cropped. Because the blots are cut prior to hybridisation with antibodies in order to reduce interference caused by polyclonal antibodies and save resources.


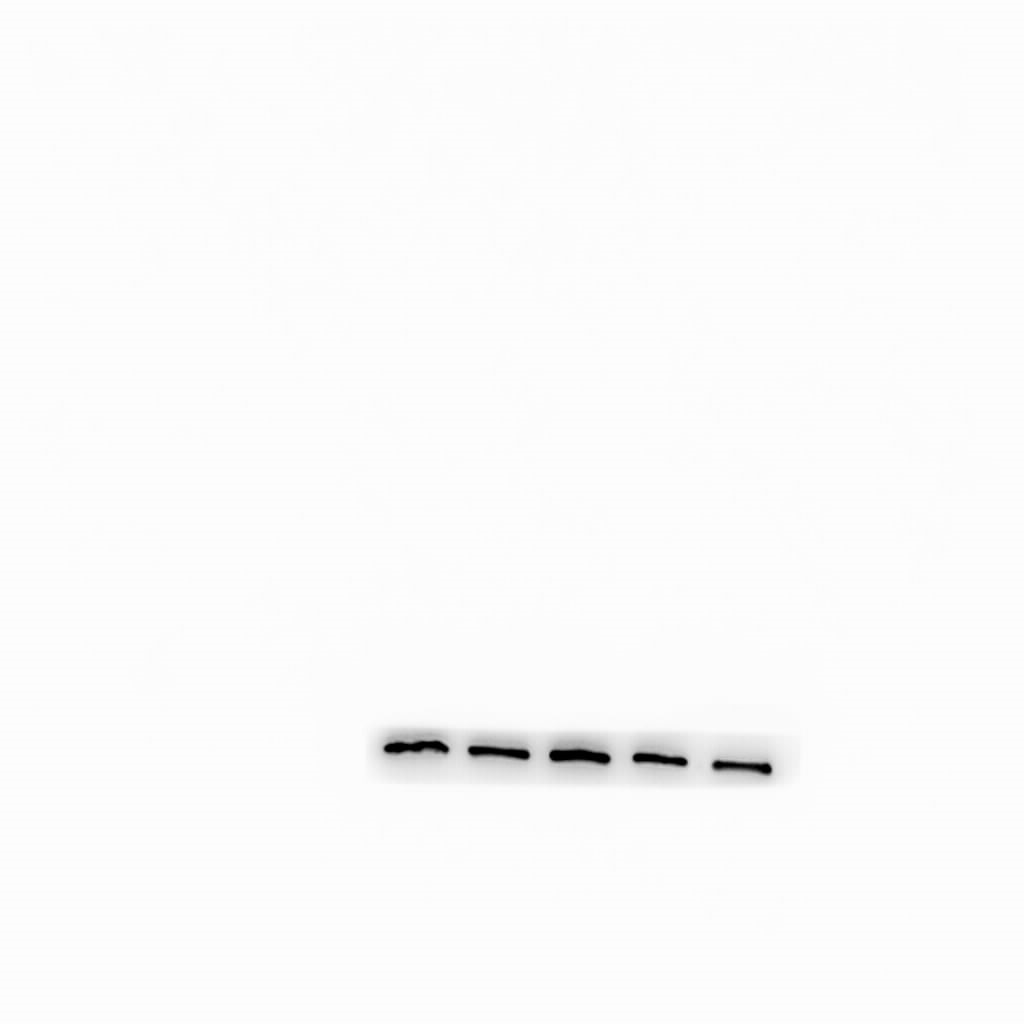


CHSP

PE

CH

GH

NP

Kir6.1 (60 kDa)


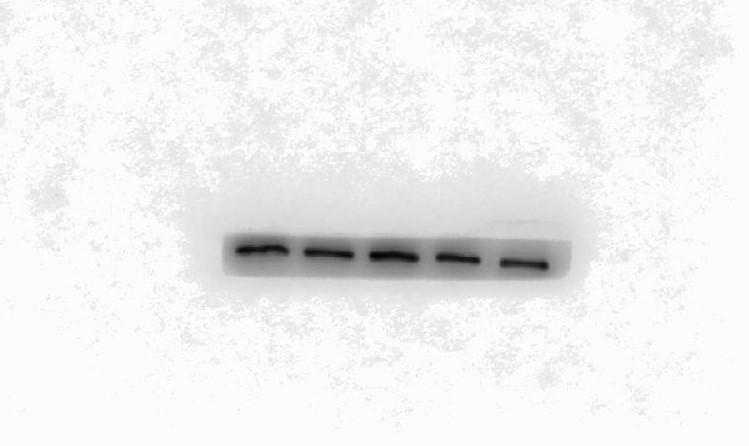
Figure 1 Imaging of the protein expression levels of Kir6.1 in patients with normal parturients (NP), gestational hypertension (GH), chronic hypertension (CH), preeclampsia (PE) and chronic hypertension with superimposed preeclampsia (CHSP). The primary antibody of Kir6.1 (Alomone, Jerusalem, Israel) is polyclonal antibody. Predicted molecular weight: 60 kDa.

After adjusting through Quantity-One, the membrane edge is clearly visible. The picture is as follows:


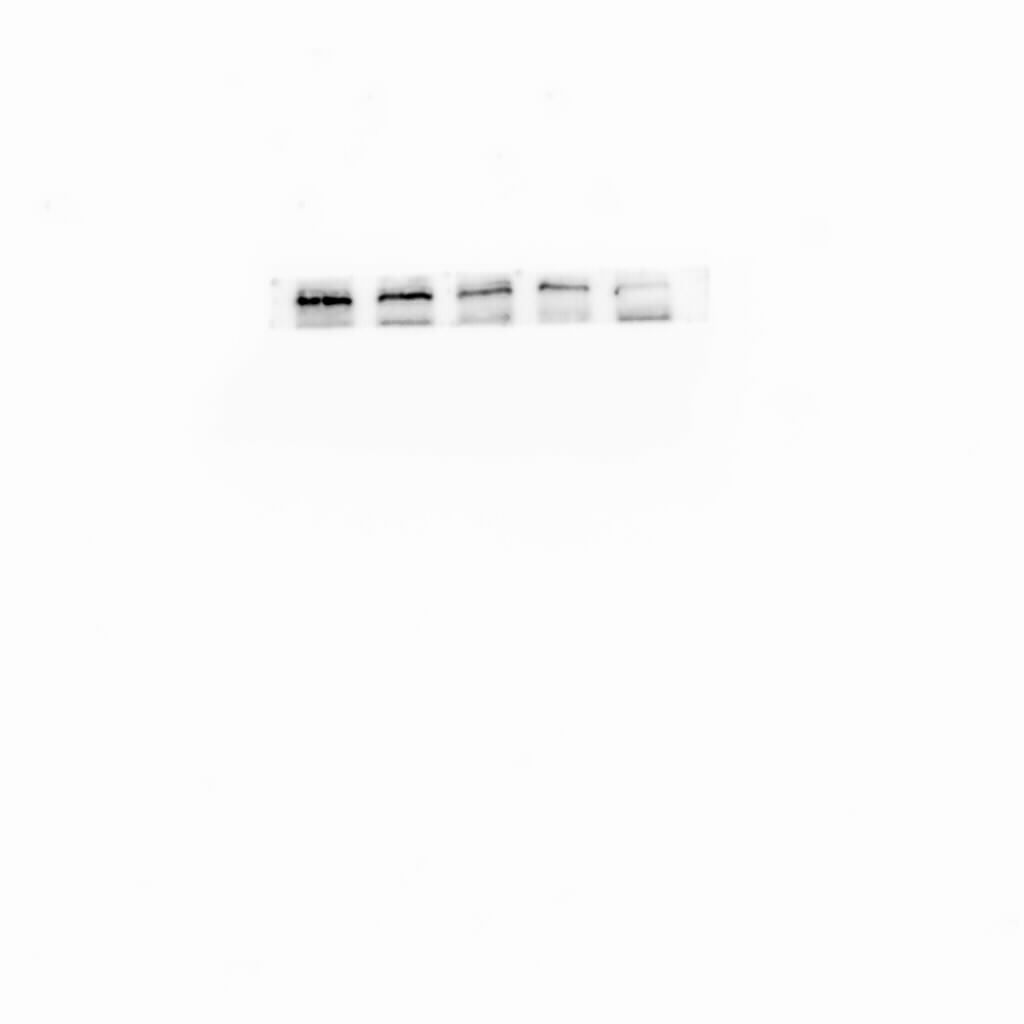


SUR2B (174 kDa)

CH

NP

GH

PE

CHSP

Figure 2 Imaging of the protein expression levels of SUR2B in patients with normal parturients (NP), gestational hypertension (GH), chronic hypertension (CH), preeclampsia (PE) and chronic hypertension with superimposed preeclampsia (CHSP). The primary antibody of SUR2B (Abcam, Cambridge, UK) is polyclonal antibody. Predicted molecular weight: 174 kDa.


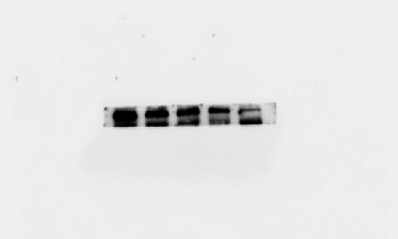
After adjusting through Quantity-One, the membrane edge is clearly visible. The picture is as follows:


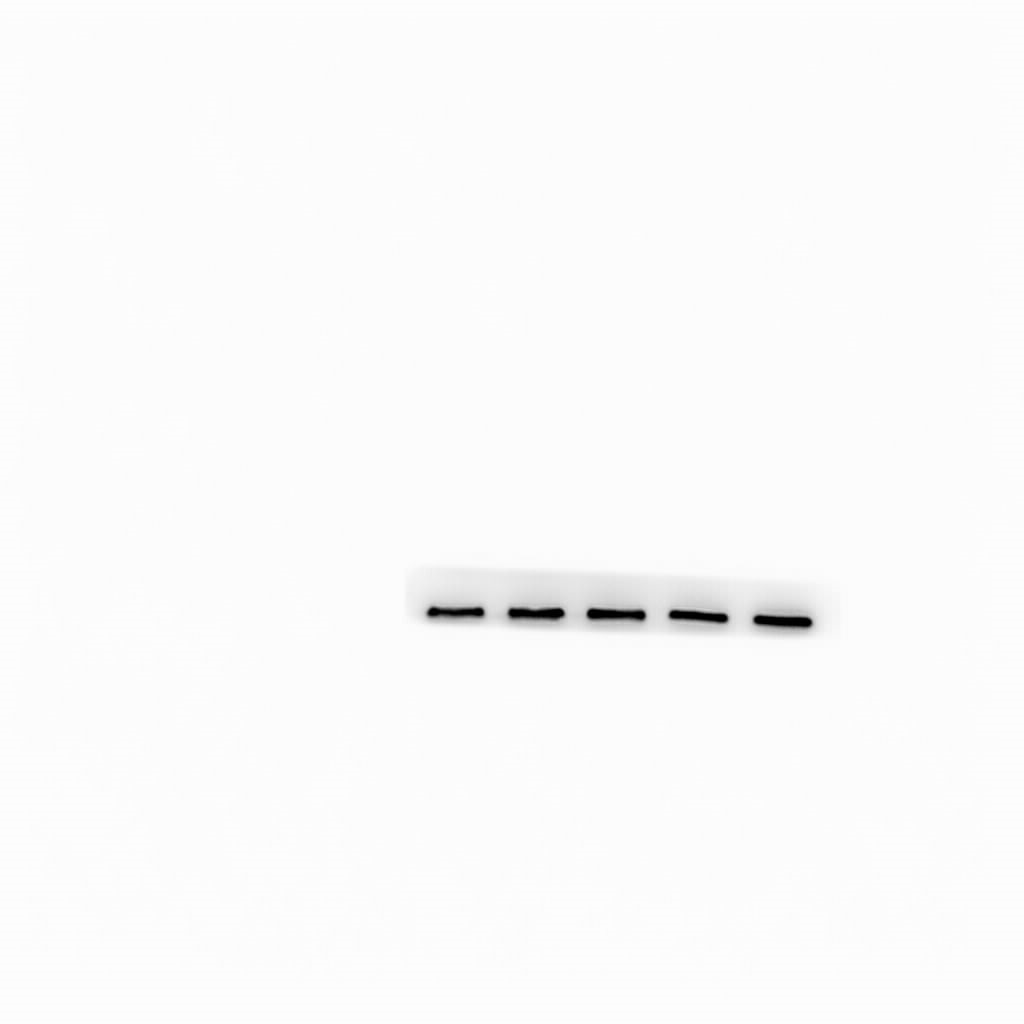


GAPDH (36 kDa)

CH

PE

CHSP

NP

GH

Figure 3 Imaging of the protein expression levels of GAPDH in patients with normal parturients (NP), gestational hypertension (GH), chronic hypertension (CH), preeclampsia (PE) and chronic hypertension with superimposed preeclampsia (CHSP). The primary antibody of GAPDH (Bioworld, Irving, TX, USA) is monoclonal antibody. Predicted molecular weight: 36 kDa.


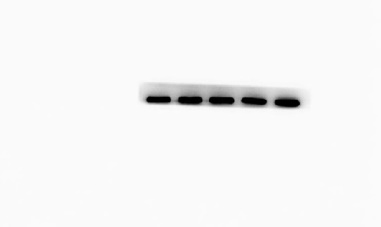
After adjusting through Quantity-One, the membrane edge is clearly visible. The picture is as follows:
